# Predictors of CD4:CD8 Ratio Normalization and Its Effect on Health Outcomes in the Era of Combination Antiretroviral Therapy

**DOI:** 10.1371/journal.pone.0077665

**Published:** 2013-10-30

**Authors:** Victor Leung, Jennifer Gillis, Janet Raboud, Curtis Cooper, Robert S. Hogg, Mona R. Loutfy, Nima Machouf, Julio S. G. Montaner, Sean B. Rourke, Chris Tsoukas, Marina B. Klein, the CANOC Collaboration

**Affiliations:** 1 Department of Pathology and Laboratory Medicine, University of British Columbia, Vancouver, Canada; 2 Toronto General Research Institute, University Health Network, Toronto, Canada; 3 Dalla Lana School of Public Health, University of Toronto, Toronto, Canada; 4 University of Ottawa, The Ottawa Hospital Research Institute, Ottawa, Canada; 5 Faculty of Health Sciences, Simon Fraser University, Burnaby, Canada; 6 British Columbia Centre for Excellence in HIV/AIDS, Vancouver, Canada; 7 Women’s College Research Institute, Women’s College Hospital, Toronto, Canada; 8 Maple Leaf Medical Clinic, Toronto, Canada; 9 Department of Medicine, University of Toronto, Toronto, Canada; 10 Clinique médicale l’Actuel, Montreal, Canada; 11 Division of AIDS, Department of Medicine, University of British Columbia, Vancouver, Canada; 12 Ontario HIV Treatment Network, Toronto, Canada; 13 Department of Medicine, McGill University, Montreal, Canada; 14 McGill University Health Centre, Montreal, Canada; Rush University, United States of America

## Abstract

**Background:**

HIV leads to CD4:CD8 ratio inversion as immune dysregulation progresses. We examined the predictors of CD4:CD8 normalization after combination antiretroviral therapy (cART) and determined whether normalization is associated with reduced progression to AIDS-defining illnesses (ADI) and death.

**Methods:**

A Canadian cohort of HIV-positive adults with CD4:CD8<1.2 prior to starting cART from 2000–2010 were analyzed. Predictors of (1) reaching a CD4:CD8 ≥1.2 on two separate follow-up visits >30 days apart, and (2) ADI and death from all causes were assessed using adjusted proportional hazards models.

**Results:**

4206 patients were studied for a median of 2.77 years and 306 (7.2%) normalized their CD4:CD8 ratio. Factors associated with achieving a normal CD4:CD8 ratio were: baseline CD4+ T-cells >350 cells/mm^3^, baseline CD8+ T-cells <500 cells/mm^3^, time-updated HIV RNA suppression, and not reporting sex with other men as a risk factor. There were 213 ADIs and 214 deaths in 13476 person-years of follow-up. Achieving a normal CD4:CD8 ratio was not associated with time to ADI/death.

**Conclusions:**

In our study, few individuals normalized their CD4:CD8 ratios within the first few years of initiating modern cART. This large study showed no additional short-term predictive value of the CD4:CD8 ratio for clinical outcomes after accounting for other risk factors including age and HIV RNA.

## Introduction

Early in the epidemic, it was recognized that HIV infection resulted in selective and dramatic CD4+ T-cell depletion [1], [2]. This loss is accompanied by an increase in CD8+ T-cells resulting in an inverted CD4:CD8 ratio [3]. CD4+ T-cell depletion is a widely recognized prognostic marker for short and long term outcomes in HIV and AIDS [4]. Today, absolute CD4+ T-cells are regularly used as an endpoint in HIV randomized controlled trials, as a criterion for starting antiretroviral therapy, and as a surrogate for the risk of developing opportunistic infections [4], [5]. However, other T-cell markers such as the percentage of CD4+ T-cells and the CD4:CD8 ratio have also been shown to predict AIDS and non-AIDS related morbidities [6]–[8].

Combination antiretroviral therapy (cART) has successfully reduced the incidence of opportunistic infections and premature mortality [9], [10]. Although cART can achieve potent and sustained viral suppression permitting normalization in CD4+ T-cell counts, this does not necessarily indicate a normal immune system. More recently, there has been a focus on immune activation as a correlate of impaired CD4+ T-cell recovery and as an independent predictor of mortality [11]. Indeed, prognostic markers for clinical outcomes in patients with fully suppressed HIV RNA are needed. Unlike the increases in CD4+T-cells, the CD4:CD8 ratio very often remains inverted in adults treated with cART for reasons that are unclear [12]. Some hypothesize that lymphocyte activation and subpopulation changes, interleukin effects, co-infections, and immunosenescence may play roles [13]. It would be expected that more HIV-positive individuals should achieve CD4:CD8 normalization in the era of effective modern cART, but the extent of ratio normalization among cART treated patients has yet to be described in a large cohort of patients.

The primary objective of our study was to determine the incidence and clinical predictors of CD4:CD8 ratio normalization after initiating cART. Our secondary objective was to determine if CD4:CD8 ratio could have an additional role as a prognostic marker for improved health outcomes with respect to developing AIDS-defining illness or death from all causes.

## Methods

### Ethics Statement

The human subjects activities of the Canadian Observational Cohort (CANOC) have been approved by the Simon Fraser University Research Ethics Board and the University of British Columbia Research Ethics Board as well as the local institutional review boards at each of the participating cohorts, as follows: Providence Health Care Research Institute Office of Research Services, The Ottawa Hospital Research Ethics Board, University Health Network Research Ethics Board, Véritas Institutional Review Board (IRB), Biomedical C (BMC) Research Ethics Board of the McGill University Health Centre, University of Toronto HIV Research Ethics Board (HIV REB), and Women's College Hospital Research Ethics Board. Local cohorts have obtained written consent except the following: HOMER (IRB approved the retrospective use of anonymous administrative data without requiring consent; an information sheet for participants is provided in lieu of a consent form); Ottawa Hospital Cohort (IRB approved the anonymous use of data retrospectively abstracted from clinical care databases without requiring consent); UHN (REB approved the anonymous use of data retrospectively abstracted from clinical care databases without requiring consent); MUHC (IRB approved the anonymous use of data retrospectively abstracted from clinical care databases without requiring consent; patients signed a general waiver on opening a medical chart at the hospital but no specific study related consent); MLMC (REB has approved the anonymous use of data retrospectively abstracted from clinical care databases without requiring consent); and EARTH (REB approved the anonymous use of data retrospectively abstracted from clinical care databases without requiring consent; patients signed a general waiver on opening a medical chart at the hospital but no specific study related consent).

### Cohort Description

CANOC included antiretroviral naïve HIV-positive individuals who initiated cART on or after January 1, 2000 from 8 participating cohorts in British Columbia, Ontario and Quebec. Individuals from each cohort were eligible for inclusion into CANOC if they met the following criteria: documented HIV infection, 18 years of age and older, and had at least 1 measurement of CD4+ T-cell count and HIV RNA within 6 months of initiating cART. Patient selection and data extraction were performed locally at the data centers of participating cohorts and pooled at the Project Data Centre in Vancouver, British Columbia. Further details of the participating cohorts and the CANOC structure are described elsewhere [14].

### Incidence and Predictors of CD4:CD8 Ratio Normalization

Participants were included in this analysis if they had at least 1 CD4:CD8 ratio measurement within 2 years prior to starting cART (defined as at least 2 classes of antiretroviral medications), had at least 2 CD4:CD8 ratio measurements after cART, and had an inverted CD4:CD8 ratio defined as <1.2 prior to initiating cART. Normalization was defined as a CD4:CD8 ratio ≥1.2 on two consecutive follow-up visits >30 days apart. Individuals who did not achieve normalization of their CD4:CD8 ratio were censored on the date of their last CD4:CD8 measurement.

### Effect of CD4:CD8 Ratio Normalization on Time to AIDS-defining Illness (ADI) or Death

ADI were defined according to the Centers for Disease Control and Prevention [15]. Participants were included in the analysis of time to ADI or death (all-cause mortality) if they were from sites with electronic ADI data and did not have an ADI prior to initiating cART. Individuals who did not have an ADI or die were censored at their date of last follow-up.

### Statistical Methods

Demographic and clinical characteristics at baseline were summarized with medians and interquartile ranges or frequency and percent. HIV risk factors were treated as binary variables and were not mutually exclusive. Kaplan-Meier (KM) survival methods were used to estimate cumulative incidence of CD4:CD8 ratio normalization from cART initiation. Univariate proportional hazard (PH) models were used to examine associations of sociodemographic and clinical variables hypothesized to be associated with normalization of CD4:CD8 ratio based on literature [12], [16]–[19]. The following variables were examined: age, gender, risk factors for HIV acquisition including men who have sex with men (MSM) and injection drug use (IDU), year of cART initiation, type of cART regimen, baseline CD4+ T-cell count and CD8+ T-cell count and time-updated HIV RNA. Analyses were adjusted for the number of CD4:CD8 measurements per year of follow-up as persons with more frequent measurements might be observed to normalize sooner.

The primary correlate of interest in the analysis of time to ADI/death was the time-updated binary indicator of CD4:CD8 normalization. KM methods for time-dependent covariates were used to examine the associations of CD4:CD8 normalization and viral load suppression with time to ADI or death [20]. PH models were used to estimate the association of CD4:CD8 normalization with time to ADI or death after adjusting for other variables hypothesized to be associated with ADIs and death based on the background literature. When covariates were considered to be collinear (e.g. IDU and hepatitis C), the variable with the strongest association was included in the multivariable model.

Sensitivity analyses were conducted to examine the effects of (1) defining normalization of the CD4:CD8 ratio as ≥1 or ≥1.5, (2) excluding individuals with inverted baseline CD4:CD8 ratios measured more than 12 months prior to starting cART, (3) excluding late presenters, defined as having an ADI/death within 3 months after cART initiation and (4) including patients with a history of ADI prior to cART initiation.

All analyses were performed using SAS software, version 9.3 (SAS Institute, Cary, NC).

## Results

A total of 5798 individuals enrolled in CANOC initiated cART between January 1, 2000 and December 31, 2010. For the time to normalization analysis, 4206 participants met the inclusion criteria. The non-mutually exclusive reasons for exclusion included: absence of CD8+ T-cell counts for calculation of the CD4:CD8 ratio (n = 309), absence of CD4:CD8 ratios at baseline (n = 870), lack of PI-based or NNRTI-based cART use (n = 142), normal CD4:CD8 ratios prior to cART (n = 28), and fewer than 2 ratio measurements in follow-up (n = 982).

In the time to ADI/death analysis, 3405 participants were included. Participants were excluded because they were from sites without electronic ADI data (n = 186) and/or because they were diagnosed with an ADI prior to initiating cART (n = 788).

The demographic and clinical characteristics of participants in the time to CD4:CD8 normalization and time to ADI/death analyses, summarized in Table 1, show that the populations were similar.

**Table 1:** Baseline characteristics of participants in the time to CD4:CD8 normalization and time to ADI/death analyses.

Variable	CD4:CD8 Normalization (n = 4206), n (%)	ADI/Death (n = 3405), n (%)
Age at cART initiation^a^	40 (34–47)	40 (34–46)
Gender		
Female	779 (19%)	644 (19%)
Male/Transgender	3426 (81%)	2761 (81%)
Province		
British Columbia	2056 (49%)	1731 (51%)
Ontario	925 (22%)	767 (23%)
Quebec	1225 (29%)	907 (27%)
HIV risk factor		
Men who have sex with men	1727 (41%)	1420 (42%)
Injection drug use	996 (24%)	832 (24%)
Heterosexual	1268 (30%)	1066 (31%)
Endemic country	378 (9%)	322 (9%)
Blood transfusion	102 (3%)	86 (3%)
Mother-to-child transmission	25 (1%)	21 (1%)
Unknown	1265 (30%)	997 (29%)
Hepatitis C co-infection		
Yes	940 (22%)	781 (23%)
No	2135 (51%)	1662 (49%)
Unknown	1131 (27%)	962 (28%)
Baseline CD4+ T-cell count^a^	190 (100–276)	200 (120–280)
Baseline HIV RNA (log_10_ copies/mL)^a^	4.9 (4.4–5.0)	4.9 (4.4–5.0)
Suppressed HIV RNA at baseline	113 (3%)	71 (2%)
Year of cART initiation^a^	2005 (2002–2007)	2005 (2002–2007)
Baseline cART		
NNRTI-based	1902 (44%)	1620 (46%)
Boosted PI-based	1946 (45%)	1530 (44%)
Single PI-based	358 (8%)	255 (7%)
CD4:CD8 measurements per year		
<3	740 (18%)	589 (17%)
3–4	946 (22%)	759 (22%)
5–6	1504 (36%)	1218 (36%)
>6	1016 (24%)	839 (25%)

a Median (interquartile range).

cART, Combination antiretroviral therapy; NNRTI, Non-nucleoside reverse transcriptase inhibitor; PI, Protease inhibitor.

### Incidence and Predictors of CD4:CD8 Ratio Normalization

**Figure 1:**
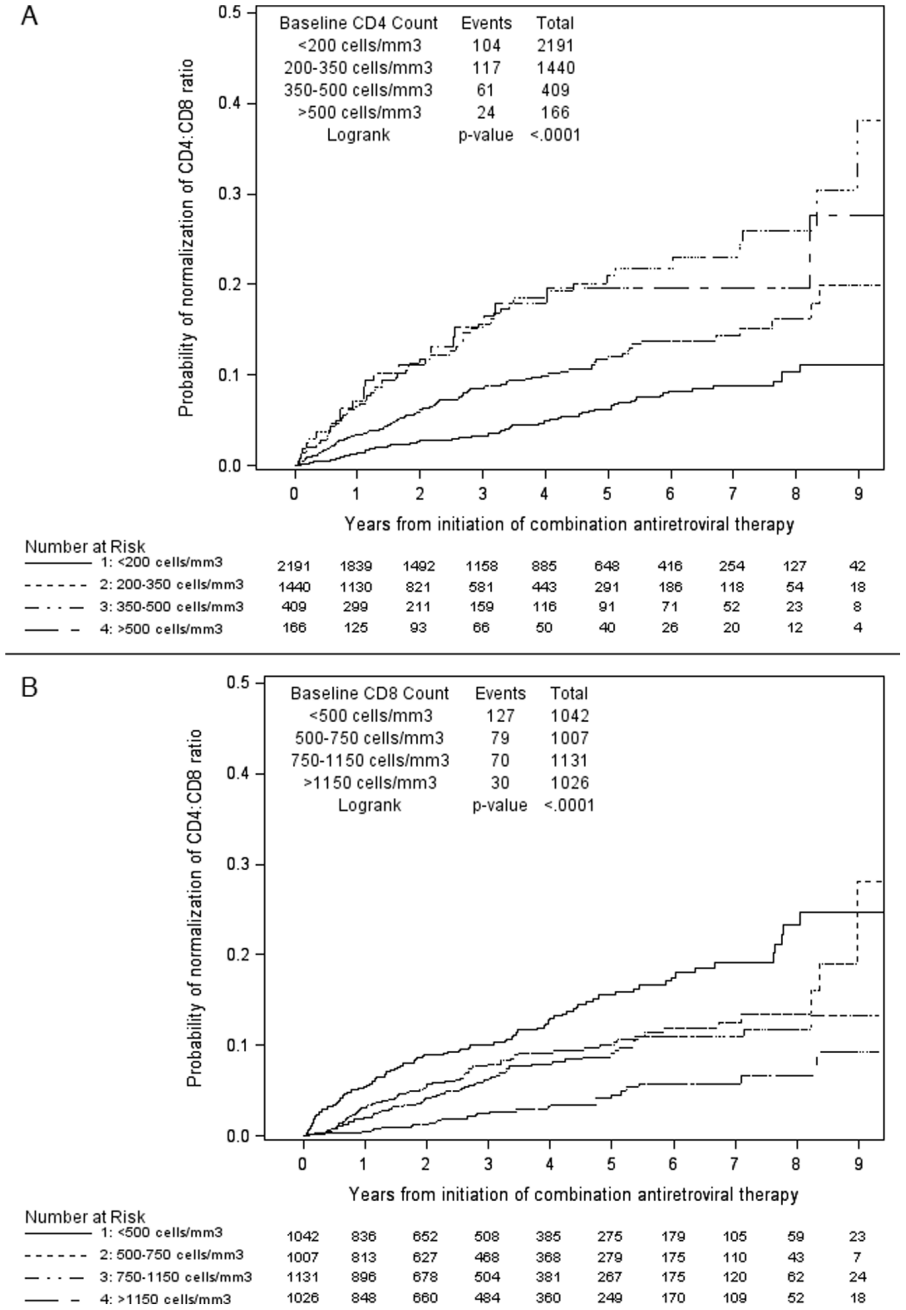
Kaplan-Meier curves of time to CD4:CD8 normalization by: (a) baseline CD4+ T-cells; (b) baseline CD8+ T-cells.

During a median follow-up of 2.77 years (14048 person-years), a total of 306 individuals (7.2%) normalized their CD4:CD8 ratio (0.022/person-year follow-up). The Kaplan-Meier curves of the time to CD4:CD8 normalization are shown in Figure 1. Individuals with baseline CD4+ T-cell counts <200 cells/mm^3^ normalized more slowly than those with higher CD4+ T-cell counts, whereas individuals with lower baseline CD8+ T-cell counts (<500 cells/mm^3^) normalized more quickly than those with higher counts. The probability of achieving a normal CD4:CD8 ratio at five years after initiation of cART was 6.1% for those with baseline CD4+ T-cells <200 cells/mm^3^ compared to 21% for those with baseline CD4+ T-cells >350 cells/mm^3^, and 16% for those with baseline CD8+ T-cells <500 cells/mm^3^ compared to 4.5% for those with baseline CD8+ T-cells >1150 cells/mm^3^. Interestingly, no plateau in normalization was apparent suggesting that normalization may continue to occur even long after cART initiation. Variables associated with time to CD4:CD8 ratio normalization in univariate and multivariable PH models are shown in Table 2. In the multivariable analysis, individuals with a higher baseline CD4+ T-cell count, lower baseline CD8+ T-cell count and time-updated HIV RNA suppression were more likely to normalize. Those reporting MSM as a risk factor were less likely to normalize than non-MSM. We observed no trend to increased rates of normalization according to calendar year of cART initiation.

**Table 2:** Univariable and multivariable proportional hazards model for time to normalization of CD4:CD8 ratio.

	n = 4206			n = 3320		
Variable	UnadjustedHazard Ratio	(95% CI)	p-value	AdjustedHazard Ratio	(95% CI)	p-value
**DEMOGRAPHICS**						
Age - continuous (per 10 years)	1.07	(0.95,1.20)	0.24	1.09	(0.95,1.24)	0.21
Gender (male/transgender vs. female)	0.75	(0.58,0.99)	0.04	0.91	(0.64,1.30)	0.61
HIV risk factor						
MSM	0.71	(0.55,0.91)	<.01	0.65	(0.47,0.90)	0.01
IDU	0.93	(0.71,1.23)	0.63	0.89	(0.64,1.24)	0.49
Endemic country	1.14	(0.78,1.65)	0.50	–	–	–
Heterosexual contact	1.25	(0.98,1.61)	0.08	–	–	–
Province						
British Columbia	1	–	–	1	–	–
Ontario	1.19	(0.89,1.59)	0.24	1.23	(0.85,1.79)	0.27
Quebec	1.36	(1.05,1.75)	0.02	1.35	(0.95,1.91)	0.09
**CLINICAL**						
Year of cART initiation						
2000–2001	1	–	–	1	–	–
2002–2003	1.01	(0.73,1.40)	0.95	0.96	(0.66,1.40)	0.85
2004–2005	1.09	(0.78,1.53)	0.62	0.82	(0.55,1.22)	0.33
2006–2007	1.25	(0.86,1.81)	0.24	1.10	(0.72,1.70)	0.65
2008–2010	1.73	(1.04,2.89)	0.04	0.74	(0.40,1.40)	0.36
Baseline cART regimen						
Single PI-based	1	–	–	1	–	–
Boosted PI-based	0.93	(0.64,1.34)	0.68	1.29	(0.82,2.02)	0.27
NNRTI-based	0.85	(0.60,1.22)	0.39	1.10	(0.72,1.69)	0.65
**LABORATORY**						
Baseline CD4+ T-cell count						
>350 cells/mm^3^	1	–	–	1	–	–
200–350 cells/mm^3^	0.54	(0.41,0.72)	<.0001	0.33	(0.24,0.45)	<.0001
<200 cells/mm^3^	0.27	(0.20,0.36)	<.0001	0.07	(0.05,0.11)	<.0001
Baseline CD8+ T-cell count						
<500 cells/mm^3^	1	–	–	1	–	–
500–750 cells/mm^3^	0.65	(0.49,0.86)	<.01	0.35	(0.25,0.49)	<.0001
750–1150 cells/mm^3^	0.53	(0.39,0.71)	<.0001	0.19	(0.13,0.28)	<.0001
>1150 cells/mm^3^	0.24	(0.16,0.36)	<.0001	0.08	(0.05,0.12)	<.0001
Baseline CD4:CD8 ratio (per 0.1)	1.48	(1.42,1.53)	<.0001	–	–	–
Baseline HIV RNA (per log_10_ copies/ml)	1.02	(0.89,1.17)	0.74	–	–	–
Time-updated HIV RNA (per log_10_ copies/ml	0.71	(0.61,0.83)	<.0001	–	–	–
Time-updated HIV RNA suppression (<50 copies/ml)	1.46	(1.10,1.94)	<.01	1.61	(1.18,2.21)	<.01
Rate of CD4:CD8 measures per year						
<3 measures	0.90	(0.63,1.30)	0.58	0.82	(0.55,1.22)	0.33
3–4 measures	1	–	–	1	–	–
5–6 measures	1.37	(1.01,1.84)	0.04	1.63	(1.16,2.29)	<.01
>6 measures	1.65	(1.17,2.33)	<.01	2.12	(1.39,3.23)	<.001

For the time to CD4:CD8 normalization, there was no difference observed between CD4+ T-cell categories of 350–500 cells/mm^3^ and >500 cells/mm^3^ at baseline, but there were relatively few individuals (n = 166) with baseline CD4+ T-cells >500 cells/mm^3^.

### CD4:CD8 Ratio Normalization and Risk of ADI/Death

**Figure 2:**
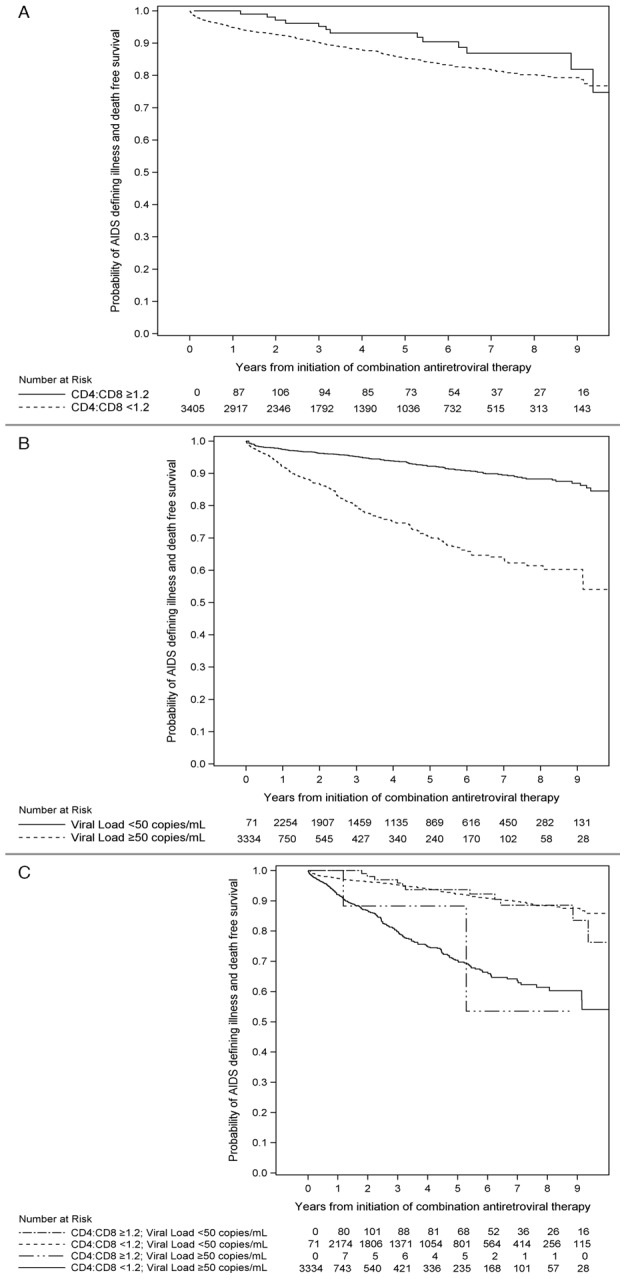
Kaplan-Meier curves of time to AIDS-defining illness or death by time updated: a) CD4:CD8 normalization; b) virologic suppression; c) stratification among 4 groups according to CD4:CD8 normalization, viral load suppression, both, and neither.

There were a total of 427 ADI/deaths observed during the study period over 13476 person-years of follow-up (0.032 events/person-year follow-up): 177 individuals were diagnosed with an ADI, 36 had an ADI and then died, and 214 individuals died without a prior ADI. KM curves were constructed to explore the association between CD4:CD8 normalization and suppressed HIV RNA with time to ADI/death (Figure 2). In these plots, individuals can move back and forth between having a normalized CD4:CD8 ratio or suppressed HIV RNA at each failure point. At cART initiation, few patients had normalized ratios or suppressed HIV RNA. Individuals were stratified into four groups depending on whether they had a normalized CD4:CD8 ratio only, had suppressed HIV RNA only, had both a normalized ratio and suppressed HIV RNA, or had neither a normalized ratio nor suppressed HIV RNA (Figure 2c). Very few patients (n = 31) normalized their ratio without also suppressing HIV RNA. The time to ADI/death did not differ between individuals who only had suppressed HIV RNA, only had a normalized ratio, or had both a normalized ratio and suppressed HIV RNA, whereas individuals who had neither a suppressed HIV RNA nor a normalized ratio were at greatest risk for ADI/death.

The variables associated with time to ADI or death in the univariate and multivariable proportional hazards models are shown in Table 3. In multivariable models, lower baseline CD8+ T-cell count and time-updated HIV RNA suppression were associated with a longer time to ADI or death. For baseline CD4+ T-cell categories, only CD4+ T-cells <200 cells/mm^3^ was significantly associated with increased risk of ADI/death. Additionally, older age and IDU risk factor were associated with a higher risk of ADI/death (Table 3). Changing the threshold for CD4:CD8 ratio normalization to 1 or to 1.5 did not impact the inferences from the multivariable model. Furthermore, modelling CD4:CD8 ratio as a continuous variable yielded similar results to the main analysis (data not shown).

**Table 3:** Univariable and multivariable proportional hazards model for time to ADI or death.

	n = 3405			n = 2746		
Variable	UnadjustedHazard Ratio	(95% CI)	p-value	AdjustedHazard Ratio	(95% CI)	p-value
Time-updated CD4:CD8 ratio >1.0	0.77	(0.51,1.16)	0.21	–	–	–
Time-updated CD4:CD8 ratio >1.2	0.84	(0.48,1.46)	0.53	0.99	(0.50,1.95)	0.97
Time-updated CD4:CD8 ratio >1.5	0.90	(0.37,2.17)	0.81	–	–	–
Age - continuous (per 10 years)	1.25	(1.14,1.38)	<.0001	1.38	(1.22,1.55)	<0.0001
Gender (male/transgender vs. female)	0.85	(0.67,1.06)	0.15	1.05	(0.79,1.39)	0.76
Province						
British Columbia	1	–	–	1	–	–
Ontario	0.56	(0.42,0.73)	<.0001	1.05	(0.76,1.46)	0.78
Quebec	0.58	(0.45,0.75)	<.0001	1.02	(0.75,1.39)	0.88
HIV risk factor						
MSM	0.52	(0.41,0.65)	<.0001	0.75	(0.55,1.03)	0.07
IDU	2.24	(1.81,2.79)	<.0001	1.38	(1.03,1.85)	0.03
Endemic country	0.55	(0.36,0.86)	<0.01	0.72	(0.43,1.19)	0.20
Heterosexual contact	0.92	(0.73,1.15)	0.45	–	–	–
Year of cART initiation						
2000–2001	1	–	–	1	–	–
2002–2003		(0.73,1.20)	0.60	1.09	(0.81,1.47)	0.57
2004–2005	0.66	(0.50,0.86)	<.01	0.83	(0.60,1.17)	0.30
2006–2007	0.51	(0.37,0.69)	<.0001	0.73	(0.50,1.07)	0.11
2008–2010	0.26	(0.14,0.48)	<.0001	0.47	(0.24,0.92)	0.03
Baseline cART regimen						
Single PI-based	1	–	–	1	–	–
NNRTI-based	0.95	(0.68,1.34)	0.78	1.21	(0.79,1.86)	0.39
Boosted PI-based	1.05	(0.74,1.49)	0.79	1.04	(0.69,1.57)	0.84
Baseline CD4+ T-cell count						
>350 cells/mm^3^	1	–	–	1	–	–
200–350 cells/mm^3^	0.92	(0.65,1.30)	0.64	0.97	(0.65,1.45)	0.89
<200 cells/mm^3^	1.70	(1.24,2.33)	<.001	1.47	(1.00,2.17)	0.05
Baseline CD8+ T-cell count						
<500 cells/mm^3^	1	–	–	1	–	–
500–750 cells/mm^3^	0.48	(0.37,0.63)	<.0001	0.66	(0.49,0.90)	<.01
750–1150 cells/mm^3^	0.47	(0.36,0.61)	<.0001	0.72	(0.52,0.98)	0.04
>1150 cells/mm^3^	0.58	(0.45,0.74)	<.0001	0.95	(0.69,1.32)	0.77
Baseline CD4:CD8 ratio (per 0.1)	0.93	(0.89,0.98)	<.01	–	–	–
Suppressed HIV RNA at baseline	0.44	(0.16,1.18)	0.10	–	–	–
Baseline HIV RNA (per log_10_ copies/ml)	1.45	(1.24,1.69)	<.0001	–	–	–
Time-updated HIV RNA (per log_10_ copies/ml)	1.74	(1.63,1.86)	<.0001	–	–	–
Time-updated HIV RNA suppression	0.22	(0.18,0.27)	<.0001	0.26	(0.20,0.33)	<.0001

Exclusion of individuals with baseline CD4:CD8 ratios obtained more than 12 months prior to starting cART did not change our results for the ADI/death analysis. However, when late presenters (those with ADI or death within 3 months of starting cART) were excluded, reducing the sample size, baseline cART regimen, CD4+ and CD8+ T-cell counts were no longer statistically significantly associated with ADI or death but the magnitude of the association of CD4:CD8 normalization with ADI/death remained the same.

When patients with an ADI prior to cART initiation were included in the analysis, we observed 142 additional clinical events in follow-up among 651 patients with sufficient CD4:CD8 ratio data and available electronic ADI data (57 ADIs, 26 ADI with subsequent death, and 59 deaths). Normalized CD4:CD8 ratio remained not associated with AIDS/death (adjusted HR 1.02, 95% CI 0.55–1.89) and inference for other covariates was unchanged.

## Discussion

In a large Canadian observational cohort of HIV+ treatment naive patients we found that only 7.2% of adults achieved a normal CD4:CD8 ratio of ≥1.2 within a median of 3 years of initiating modern cART, suggesting that the vast majority of successfully treated HIV-positive patients remain immunologically impaired. However, failure to achieve normalization did not appear to result in poorer clinical outcomes in the presence of controlled HIV at least over the short-term. Time-dependent normalized CD4:CD8 ratio was not associated with a lower risk of ADI/death after adjusting for covariates. Instead, we found that older age, IDU, CD4+ T-cell count <200 cells/mm^3^, higher pre-treatment CD8+ T-cells and time-updated HIV RNA suppression were associated with ADIs/death.

Understanding why immune dysfunction persists despite apparent effective therapy and what impact persistent immune dysfunction has on clinical events is important. Furthermore, many of the complications that patients are currently experiencing likely relate at least in part to chronic inflammatory processes. It is therefore of interest to determine the utility of immune prognostic markers other than the absolute CD4+ T-cell count which frequently normalizes with effective cART. One such potential marker is the CD4:CD8 ratio which recently has been shown to be independently associated with T-cell activation among HIV-positive persons on suppressive cART [21]. In infants, CD4:CD8 ratio has been found to be a more sensitive predictor of HIV natural history than the absolute CD4+ T-cell count [22]. The CD4:CD8 ratio independently predicts mortality in untreated HIV in low-income countries and has also been found to be an independent predictor for developing the immune reconstitution inflammatory syndrome [23].

Not surprisingly, we found that the pre-treatment CD4+ T-cell count was the strongest predictor of failing to normalize the CD4:CD8 ratio. Participants with CD4+ T-cells <200 and 200–350 cells/mm^3^ were less likely to normalize their ratios than participants with CD4+ T-cells 350–500 cells/mm^3^. However, we could not demonstrate any additional benefit on ratio normalization in participants initiating cART at CD4+ T-cells >500 cells/mm^3^; the curves for these two categories were almost superimposable during the first 6 years of follow-up (Figure 1a), although we had limited power to estimate normalization in the higher CD4+ T-cell category. The association of higher baseline CD4+ T-cell counts and lower baseline CD8+ T-cell counts with normalization of the CD4:CD8 ratio, suggests that immune system disruption before cART predicts subsequent immune dysregulation [24], [25].

CD4+ and CD8+ T-cell counts tend to change progressively and in opposite directions in HIV infection [26]. T-cell subpopulation homeostasis is maintained by complex regulatory mechanisms that are still not fully understood [27]. Whether factors that have been suggested to influence CD4+ T-cell subsets (such as increased apoptosis of uninfected cells, a lack of redistribution from secondary lymphoid tissues, a higher undetectable residual viremia, a poor proliferate capacity of peripheral CD4+ T-cells, a reduction in thymic function) also affect ratio normalization, remains unknown at this time [28].

Interestingly, we found that individuals who failed to normalize had higher CD8+ T-cell counts at baseline. The mechanism of this difference is unexplained but may reflect differences in the control of CD8+ T-cells when compared with the control of CD4+ T-cells, as well as differences in homeostasis of CD8+ T-cell subpopulations [13], [29], [30]. The mechanisms controlling CD8+ T-cell homeostasis may obscure, delay or impede CD8+ T-cell responses following cART initiation and hence, prevent normalization of the CD4:CD8 ratio despite independent increases in the CD4+ T-cells. It has been proposed that homeostasis of total T-cells in the peripheral blood occurs by replacement with either CD4+ or CD8+ T-cells as CD4+ T-cells are depleted [31].This “blind T-cell homeostasis” theory has been challenged [26] where the mechanisms controlling this homeostasis in HIV infection may fail. In the end, the factors controlling homeostasis of T-cells, and the best marker of altered homeostasis, have yet to be elucidated.

MSM had a lower likelihood of normalization when compared with other risk factors. One potential explanation for this finding is that cellular responses against foreign antigens such as bacterial and viral agents responsible for sexually transmitted infections, such as cytomegalovirus (CMV) and syphilis, which are more common among MSM may increase immune activation and result in persistent expansion of CD8+ T-cell populations. For example, CMV-specific T-cell responses are 3–5 fold higher in HIV-positive individuals and it is estimated that 20–25% of residual immune activation in HIV is due to CMV-specific T-cell responses [32], [33]. However, to our knowledge, there are no data to clearly show that an increase in CMV-specific CD8+ T-cells translates to a decreased CD4:CD8 ratio. The role of other co-infecting pathogens that may have a higher incidence among MSM has not been fully explored.

Better virologic control was strongly associated with normalization of the CD4:CD8 ratio. Individuals with suppressed HIV RNA were 1.6 times more likely to normalize CD4:CD8 ratios. This observation is consistent with the idea that viral suppression must precede immune recovery.

To our knowledge, only one published case control study has examined factors associated with CD4:CD8 ratio normalization [19]. In this study of 160 patients, 6% were able to normalize their ratio. CD4+ T-cells >350 cells/mm^3^ and CD4:CD8 ratio of >0.5 at baseline was associated with ratio normalization. However, this study did not account for pre-treatment viral load. A larger retrospective study from Lisbon was recently presented. Of 1750 patients on cART, 6% of the cohort normalized their CD4:CD8 ratio (>1.0) in a mean time of 69 months. However, the predictors and the consequences of normalization were not reported [34]. In a more recent cohort study which aimed to characterize the recovery of multiple T-cell parameters after viral suppression, CD4:CD8 ratio was incorporated into a multiparametric measure of T-cell recovery which also included absolute CD4+ T-cells and CD4+ T-cell percentage [35]. Although the majority of this cohort reached a CD4+ T-cell count >500 cells/mm^3^, only a fraction of individuals were able to normalize the multiparametric measure of T-cell recovery. This finding supports the concept that reconstitution of CD4+ T-cell counts does not always reflect normalization of T-cell homeostasis. However, this study was too small (n = 352) to examine the clinical importance of the surrogate multiparametric measures of T-cell homeostasis.

Our group has also explored the incidence and predictors of a multiparametric measure of T-cell homeostasis designated as the T-cell phenotype (TCP) [36]. In addition to the CD4:CD8 ratio, the TCP included CD4+ T-cell count and percent, CD3+ T-cell percent, and CD8+ T-cell count and percent. Of 4459 individuals studied, only 85 individuals (2%) reached the primary endpoint of normalizing all components of the TCP. Taken together, it is clear that virologic-immune discordance is not restricted to CD4+ T-cells and that HIV infection is associated with other changes in the T-cell compartment that are not fully restored by cART in many individuals.

We chose to define a normal CD4:CD8 ratio as ≥1.2, based on a previous study that determined this to be the minimum critical value [17] and data from the Multicenter AIDS Cohort Study (MACS) which found that the baseline population CD4:CD8 ratio in HIV uninfected persons was 1.2 [37]. Other studies have used a cut-off ratio of 1.0 [19], [38]. In sensitivity analyses using cut-offs of 1 and 1.5, we found no significant differences in the predictors of normalization. Nonetheless, a “normal” CD4:CD8 ratio remains poorly defined. For example, as many as 20% of samples from adults who were felt to be healthy Caucasian subjects had CD4:CD8 ratios <1 in a 1991 study aimed at determining lymphocyte subset reference ranges [17]. Furthermore, racial differences can affect the ratio [39] and it has been proposed that the balance between CD4+ and CD8+ T-cells may be genetically controlled [40], [41]. We were not able to control for ethnicity as this data was unavailable for many contributing centres.

There are limitations to our study. The mechanisms underlying poor immunologic outcomes during therapy were not studied here, nor are they immediately evident. We could not determine if the changes in CD8+ T-cells were due to changes in activated CD8+ T-cells since we did not have surface markers for activation antigens (e.g. HLA-DR and CD38) reported on all individuals. It has been suggested that CD8+ T-cells expressing high levels of activation antigens are predictive of the decline in CD4+ T-cells and persistence of viral replication in vivo [42]–[45]. One of the limitations of using the measurements for lymphocyte counts and ratios in multicentre cohorts is the inherent variance in the measurement techniques for CD4:CD8 ratios across centres. Even with flow cytometry, the differences in instruments, antibodies, procedures and techniques that are different at the various centers participating in this study, can potentially introduce sources of variation [46]. Furthermore, we did not have information on race, hepatitis B co-infection status, CMV or other co-infections and immunologic diseases that may have influenced the CD4:CD8 ratio.

In the era of cART and durable treatment-associated viral suppression, it remains unclear how to best define immunological success. Failure to restore immunocompetence has been linked to an increased risk of morbidity and mortality associated with conditions not previously thought to be AIDS related, including cardiovascular disease, liver disease and cancers such as Hodgkin’s lymphoma [6], [13], [47], [48]. We have examined the potential role of the CD4:CD8 ratio as a marker of immune status, in particular as a measure of immune recovery on cART. We found that CD4:CD8 ratio normalization occurs rarely; even after several years of cART. Failure to normalize CD4:CD8 ratio was not associated with an increased risk for ADIs/death provided HIV was well controlled. Future studies should determine if this marker of persistent immune dysfunction is relevant to non-AIDS outcomes such as risk for cardiovascular disease and cancer.
